# Effect of a Prize-Linked Savings Intervention on Savings and Healthy Behaviors Among Men in Kenya: A Randomized Clinical Trial

**DOI:** 10.1001/jamanetworkopen.2019.11162

**Published:** 2019-09-13

**Authors:** Ellen Moscoe, Kawango Agot, Harsha Thirumurthy

**Affiliations:** 1 Department of Medical Ethics and Health Policy, Perelman School of Medicine, University of Pennsylvania, Philadelphia; 2 Center for Health Incentives and Behavioral Economics, University of Pennsylvania, Philadelphia; 3 Impact Research and Development Organization, Kisumu, Kenya

## Abstract

**Importance:**

Interventions to reduce men’s alcohol use and risky sexual behaviors are essential for reducing new HIV infections in high-prevalence settings in sub-Saharan Africa. Prize-linked savings accounts can motivate savings and may decrease expenditures on risky behaviors, but few studies have examined the HIV prevention potential of such savings interventions among men.

**Objective:**

To evaluate the effect of prize-linked savings accounts on savings behavior and expenditures on alcohol, gambling, and transactional sex among men in Kenya.

**Design, Setting, and Participants:**

Randomized clinical trial among communities in Siaya County, Kenya. Participants were men 21 years or older who owned a mobile phone, were engaged in fishing or transportation sector work, and were willing to open an account with a local bank; they were screened for eligibility between September 3 and October 5, 2018.

**Interventions:**

Eligible participants were offered savings accounts endowed with 1000 Kenya shillings (US $10) and randomized (1:1) to receive weekly lottery-based rewards contingent on growth in savings balance or to a control group that received standard interest.

**Main Outcomes and Measures:**

The primary outcome was an indicator of whether a participant saved any money in the bank account (intent-to-treat analysis) during the study period. Secondary outcomes included total amount saved in the bank account, total amount saved in all sources, and expenditures on alcohol, gambling, and transactional sex.

**Results:**

A total of 425 men were screened, 329 (77.4%) met eligibility criteria, 300 (70.6%) were enrolled (with 152 randomized to the intervention group and 148 to the control group), and 270 of 300 (90.0%) opened bank accounts. Participants’ mean age was 33.7 years (interquartile range, 13.5 years), 84.3% (253 of 300) were married, and the mean weekly earnings were US $30 (interquartile range, US $23). During a mean (SD) follow-up of 9 (2) weeks, 37.3% (50 of 134) in the intervention group saved money in a bank account vs 27.2% (37 of 136) in the control group, although the difference was not statistically significant (odds ratio, 1.62; 95% CI, 0.96-2.74). The intervention group had higher growth in bank savings balances (US $10.26; 95% CI, US $5.00-US $58.20 vs US $4.87; 95% CI, US $0.67-US $9.00) and higher total savings from all sources (US $201; 95% CI, US $133-US $269 vs US $145; 95% CI, US $88-US $202), but neither difference was statistically significant. The intervention did not have a significant effect on alcohol, gambling, and transactional sex expenditures.

**Conclusions and Relevance:**

Prize-linked savings accounts modestly increased savings among high-risk men in Kenya over a 9-week period, but the difference compared with standard-interest savings accounts was not significant. Testing of more powerful savings products is needed to assess whether such savings-led interventions can reduce men’s expenditures on alcohol, gambling, and transactional sex.

**Trial Registration:**

Social Science Registry identifier: AEARCTR-0003224, and ClinicalTrials.gov identifier: NCT04013295

## Key Points

**Question:**

Do prize-linked savings accounts lead to increased savings and reduced spending on alcohol, gambling, and transactional sex among men in Kenya?

**Findings:**

In this randomized clinical trial of 300 men, 37.3% who received a prize-linked savings intervention saved money in a bank account compared with 27.2% in the control group, although the difference was not significant. There were no significant differences in expenditures on alcohol, gambling, and transactional sex.

**Meaning:**

Prize-linked savings interventions can increase savings among men but did not significantly differ from standard-interest savings accounts; more evidence is needed on their potential for reducing spending on risk behaviors.

## Teaser

This randomized clinical trial evaluates the effect of prize-linked savings accounts, compared with standard-interest savings accounts, on savings behavior and expenditures on alcohol, gambling, and transactional sex among men in Kenya.

## Introduction

Despite a large decline in new adult HIV infections in sub-Saharan Africa (SSA) since 2010, progress has slowed in recent years, and HIV risk among adolescent girls and young women in particular remains high.^1^ Transactional sex, or the exchange of material support or goods in noncommercial sexual relationships that are often age disparate, is among the main driving factors for the HIV risk in this population.^2,3^ Although various structural and biomedical HIV prevention interventions have targeted women, there is a large gap in interventions targeting men, particularly those who engage in transactional sex.

In Kenya’s Nyanza region, where HIV prevalence is considerably higher than in other parts of the country, transactional sex, venue-based commercial sex work, and alcohol consumption^4,5,6^ are important contributors to the spread of HIV. As in other parts of eastern and southern Africa, policy responses have not adequately targeted these upstream HIV risk behaviors, particularly among men. When men are targeted with HIV prevention services, the focus has been directed on increasing their uptake of voluntary medical male circumcision and HIV testing and on linking those who are HIV positive to treatment. Few interventions have sought to reduce men’s participation in transactional sex and curtail their alcohol consumption, which is known to be a risk factor for HIV.^7,8^

As access to banking services and financial inclusion increases in SSA, there is growing potential to examine whether savings-led interventions can encourage men to save money and thereby divert spending away from alcohol and transactional sex. A recent cross-sectional study^9^ in Uganda found that men and women who saved money were less likely to have problematic alcohol use and engage in risky sexual behavior, suggesting that providing income-earning men with opportunities to save money may be one way to reduce risky behaviors. However, few studies have examined this possibility using experimental designs.

Prize-linked savings accounts are an increasingly popular way to promote savings and have been offered by credit unions, employers, and banks in several countries.^10,11^ They offer savers random, lottery-like rewards as interest income. Unlike traditional interest, which tends to be small, prize-linked savings accounts include a low probability of earning large amounts of interest income. The probability of winning prizes is usually determined by the amount of new deposits made in a given period, meaning that individuals’ chances of winning a prize increase as they save more. Offering prize-linked savings has been shown to lead to take-up of savings accounts and to modestly increase savings in the United States, South Africa, and Mexico.^12,13,14,15^ Evidence from the United States also demonstrates that having a prize-linked savings account displaces gambling,^12,16^ suggesting that it may fulfill a desire for risk taking that could apply to other risk behaviors. Therefore, prize-linked savings opportunities may be particularly appealing for men who engage in risky behaviors. However, to our knowledge, no studies to date have examined the effect of offering prize-linked savings accounts on health behaviors of high-risk men. Responding to the vital need for interventions that target men’s health behaviors in SSA, we conducted a randomized clinical trial to explore the potential for a prize-linked savings intervention to increase men’s savings and decrease their spending on alcohol, gambling, and transactional sex.

## Methods

### Study Design

**Figure 1.**
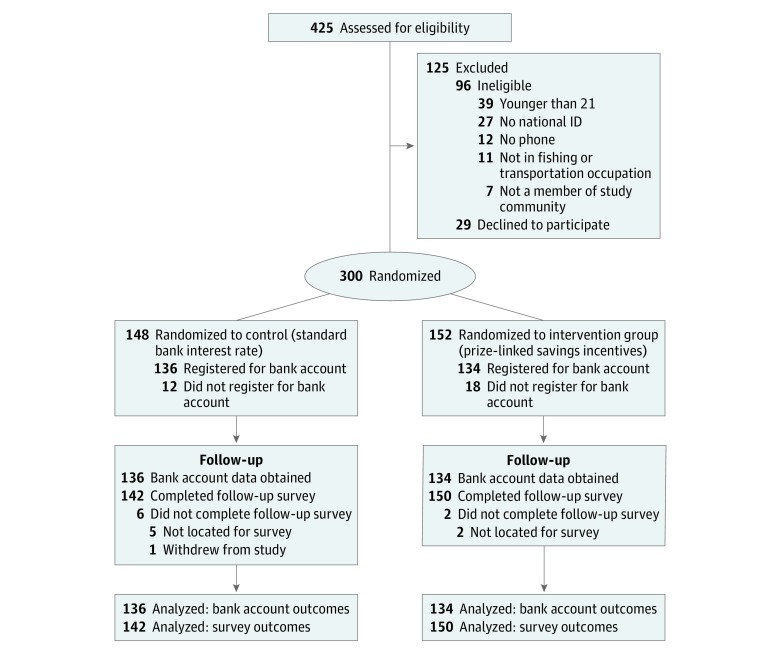
CONSORT Flow Diagram CONSORT indicates Consolidated Standards of Reporting Trials; ID, identification number.

This randomized clinical trial focused on income-earning adult men in a high HIV prevalence setting and consisted of a 9-week intervention period. All participants were given an opportunity to open a bank account that was endowed with 1000 Kenya shillings (US $10). Participants were randomized to a control group that received standard interest or to an intervention group that also received prize-linked savings rewards. Bank account balances were monitored every week, and health and savings behaviors were assessed at the beginning and end of the study. Both groups received weekly text message reminders to save money. This study followed the Consolidated Standards of Reporting Trials (CONSORT) reporting guideline (Figure 1) and was registered in the Social Science Registry (AEARCTR-0003224) and retrospectively registered on ClinicalTrials.gov. The trial protocol is available in Supplement 1.

This study was approved by the University of Pennsylvania Perelman School of Medicine Institutional Review Board and the Maseno University Ethics Review Committee. Participants provided written informed consent after passing a screening questionnaire to determine eligibility. Participants also provided written consent for the bank partner to release weekly savings balances to the study team.

### Study Setting and Population

The study was located in communities bordering Lake Victoria and semiurban market centers in Siaya County, Kenya, where the adult HIV prevalence is 21%.^17^ The study was conducted among men because it focused on the demand side of the transactional sex market. We sought to enroll men engaged in fishing and transportation, who are likely to be earning income on a regular basis. Frequent earnings may be more strongly associated with problematic alcohol use and higher participation in transactional sex, but they also make it possible for the men to save regularly. We partnered with a bank that had branches throughout Kenya, including one in the study area.

Men residing in study communities were eligible to participate if they were 21 years or older, owned a mobile phone, reported a primary or secondary occupation in the fishing or transportation sectors, and were willing to open a bank account with the banking partner. Study staff conducted a series of community meetings to explain key elements of the study. In beach communities on Lake Victoria, these meetings were coordinated with the Beach Management Unit, a local governing body that regulates fishing activities in each beach location. In the urban center, meetings were held with each motorbike taxi stand, which acts as an organizational unit for the drivers. In beach communities, men were approached by research assistants if they were on a list of fishermen currently registered to fish from that beach or if they were at the beach at the time of recruitment and appeared to be engaged in the fishing industry and be age eligible. In the urban centers, research assistants mapped all motorbike taxi stands and began recruitment at the most central stands, working their way toward the outskirts of the city until target sample sizes were reached. Those who met eligibility criteria and provided written informed consent were enrolled in the study.

### Randomization

After a baseline survey in which participants’ demographic, health, and economic characteristics were assessed, participants were randomized (1:1) to the control group or intervention group. Randomization was performed using a computer-generated algorithm, and the study group assignment for each participant identification number was preloaded on tablet computers that were used for data collection. Research assistants were masked to study group assignment until the end of the baseline survey, when the survey software (Open Data Kit) automatically routed them to deliver either the control or intervention group script based on the participant identification number. Research assistants and study staff were not masked to intervention assignment in subsequent interactions with participants.

### Bank Account Registration

Participants were given a flyer containing key information about the bank accounts. Staff from the study and banking partner helped participants register with the Kenya Revenue Authority and open bank accounts. Once their accounts were open, participants’ accounts were endowed with 1000 Kenyan shillings (US $10). The endowment amount was determined by local investigators and study staff to be locally appropriate while also providing sufficient motivation to open a bank account and not unduly influencing study participation. Research assistants distributed information about how to make deposits and check account balances, and they ensured that there was at least 1 mobile agent in each study area affiliated with the partner bank to enable participants to make deposits without traveling to the main bank branch. Participants with accounts at other banks were eligible to enroll and open accounts with the study.

### Intervention

Participants in the intervention group were eligible to receive rewards on a weekly basis if they saved any money in their account during that week. Weekly savings were defined as the increase in account balance from the previous week, net of any winnings or interest payments. Participants who saved any money (ie, >US $0) had a 20% chance of winning a small prize equivalent to 20% of their savings during that week. They also had a 2% chance of winning a large prize equivalent to 100% of their savings during that week. Prize levels were determined by local study staff and investigators. Lottery winners were selected at random by assigning a random number to eligible participants and allocating the large prize to the 2% smallest random numbers and allocating the small prize to the next 20%. The selection of prize winners was done by an investigator at the University of Pennsylvania (E.M.) each week using the runiform package in Stata (version 15; StataCorp LP). Prizes were deposited into participants’ accounts, and winners were notified by text message.

### Outcomes

Data on study outcomes were obtained from bank account balances, the baseline survey, and a follow-up survey that was administered to participants at the end of the study. We assessed several prespecified outcomes. The primary outcome was a binary variable indicating whether a participant’s savings balance increased over the study period, net of any prize winnings. Other outcomes we assessed included the total amount saved, net of any prize winnings, during the study period. These outcomes were assessed using account balance data from the bank. Because the administrative data captured savings in the study accounts only, we also assessed change in total savings, as measured by survey questions that accounted for all forms of savings in both formal and informal sources, such as savings groups, mobile banking platforms, and cash savings kept at home.

We used survey data from the beginning and end of the intervention period to measure expenditures on the following 3 risk behaviors: alcohol, gambling, and transactional sex (defined as having exchanged any money or other goods for sex). Each of these 3 risk behaviors was examined as binary variables indicating any participation in the behavior, as well as continuous variables indicating total expenditures in the past week (alcohol) or month (gambling or transactional sex). We also measured expenditures on food and nonfood items to assess effect of the intervention on total expenditures. Most survey questions were based on World Bank Living Standards Measurement Surveys^18^ and adapted to the study setting.

### Statistical Analysis

We performed *t* tests (or tests of proportions) and adjusted linear (or logistic) regressions to estimate effects of the intervention. We calculated the risk difference for the binary outcome of any savings in the study account over the study period. The adjusted regression models included controls for participants’ age, marital status, education (less than complete primary vs complete primary and higher), occupation (fishing or transportation), weekly earnings, and preexisting bank savings.

Using baseline and endline data for survey-based outcomes, we also estimated difference-in-differences regression models to measure intervention effects. These models compare the change in the outcome from baseline to endline between control and intervention groups by including individual-level fixed effects and an interaction of indicators for intervention group and survey wave. We used linear models for the difference-in-differences models for both binary and continuous outcomes due to their unbiased properties^19,20^ and ease of interpretation. In some models, we used transformed versions of the continuous savings variables to account for the skewed distribution of savings variables. We transformed the data using the inverse hyperbolic sine transformation, which is commonly used for wealth and savings data.^21^

Finally, we conducted post hoc subgroup analyses for the following 4 important subgroups of participants: those who reported any transactional sex in the past 6 months at baseline, those who reported no bank savings at baseline, those whose occupation was in the fishing sector, and those whose occupation was in the transportation sector. Analyses were limited to the 270 participants who opened bank accounts (for the bank account balance data) and the 292 participants who completed follow-up surveys (for the survey-based outcomes). The intent-to-treat analysis of bank account outcomes is summarized in eTable 1 in Supplement 2.

Given the lack of preliminary data on likely savings behavior in the control group and intervention effectiveness, power calculations were based on varying assumptions regarding the primary outcome in the control group. Assuming that 10% of the control group would increase their savings balance during the study, with a sample size of 200 there was 80% power to detect at least a 15–percentage point difference between the control and intervention groups (2-sided α = .05). If 50% of the control group saved, with 200 participants there would be 80% power to detect an increase of at least a 19–percentage point difference between the control and intervention groups. We increased the sample size to 300 with additional funding, which allowed us to detect a similar effect size (16%) if a higher proportion (50%) of the control group increased their savings balance.

## Results

Between September 3 and October 5, 2018, we screened 425 men, of whom 329 (77.4%) met eligibility criteria and 300 (70.6%) consented and were enrolled in the study (148 randomized to the control group and 152 randomized to the intervention group). The most common reason for ineligibility was age younger than 21 years (n = 39). Among 300 participants, 270 (90.0%) (136 control and 134 intervention) opened accounts with the partner bank. We conducted follow-up interviews with 292 of 300 participants (97.3%) (142 control and 150 intervention), including 267 (98.9%) of those who opened bank accounts. Participants’ bank accounts were monitored for a mean (SD) of 9 (2) weeks after they were opened, and follow-up interviews were conducted a mean (SD) of 12 (1) weeks after enrollment. One participant withdrew from the study before the follow-up survey.

Participants’ demographic and economic characteristics were similar across control and intervention groups. Participants’ mean age was 33.7 years (interquartile range [IQR], 13.5 years), 84.3% (253 of 300) were married, and their occupations were evenly divided between fishing (51.3% [154 of 300]) and transportation (48.7% [146 of 300]) (Table 1). Participants had a mean weekly earnings of US $30 (IQR, US $23), which were higher among fishermen (US $34) than among the transportation workers (ie, motorbike taxi drivers) (US $26). Almost 70% (197 of 300) of respondents did not have any savings in a bank at baseline. Respondents reported a mean of US $93 (IQR, US $88) in formal and informal savings. Almost all participants (97.7% [293 of 300]) had an account with the mobile money platform MPesa.

**Table 1.** Participant Characteristics

Variable	No. (%)			*P* Value^a^
	Full Sample (N = 300)	Control (n = 148)	Intervention (n = 152)	
**Demographics**				
Age, mean (IQR), y	33.7 (13.5)	33.6 (12.5)	33.8 (13.5)	.88
Married	253 (84.3)	123 (83.1)	130 (85.5)	.57
Has children	260 (86.7)	128 (86.5)	132 (86.8)	.93
Educational level				
Less than complete primary	92 (30.6)	47 (31.8)	45 (29.6)	.69
Complete primary or some secondary	149 (49.7)	69 (46.6)	80 (52.6)	.30
Complete secondary or higher	73 (24.3)	38 (25.7)	35 (23.0)	.59
Occupation				
Fishing	154 (51.3)	78 (52.7)	76 (50.0)	.64
Transportation	146 (48.7)	70 (47.3)	76 (50.0)	.64
Earnings in past wk, US $				
Mean (IQR)	30 (23)	26 (21)	33 (25)	.10
Occupation: fishing, mean (IQR)	34 (27)	27 (23)	40 (35)	.12
Occupation: transportation, mean (IQR)	26 (20)	25 (20)	27 (20)	.47
**Savings Behaviors**				
Has savings in a bank account	103 (34.3)	46 (31.1)	57 (37.5)	.24
Has MPesa account	293 (97.7)	143 (96.6)	150 (98.7)	.24
Keeps savings at home	120 (40.0)	53 (35.8)	67 (44.1)	.14
Belongs to a savings group	195 (65.0)	99 (66.9)	96 (63.2)	.50
Total savings, self-report, mean (IQR), US $	93 (88)	92 (84)	94 (90)	.94
**HIV Risk Behaviors**				
Any alcohol bought in past wk	82 (27.3)	48 (32.4)	34 (22.4)	.05
Alcohol expenditure in past wk, mean (IQR), US $	2 (2)	2 (3)	2 (0)	.57
Ever gambled	160 (53.3)	74 (50.0)	86 (56.6)	.25
Gambling expenditure in past mo, mean (SD), US $	16 (18)	14 (13)	17 (19)	.50
Exchanged sex in past 6 mo	142 (47.3)	69 (46.6)	73 (48.0)	.81
Total value exchanged for sex in past mo, mean (SD), US $	8 (8)	8 (10)	8 (8)	.96
Total value exchanged for sex in past mo if >US $0, mean (SD)	20 (20)	19 (19)	20 (15)	.79
≥2 Self-reported partners in past mo	122 (40.7)	56 (37.8)	66 (43.4)	.32
Self-reported risk of acquiring HIV in next y				
None	40 (13.3)	21 (14.2)	19 (12.5)	.67
Low	91 (30.3)	46 (31.1)	45 (29.6)	.78
Moderate	60 (20.0)	25 (16.9)	35 (23.0)	.19
High	91 (30.3)	51 (34.5)	40 (26.3)	.13
Not applicable	18 (6.0)	5 (1.6)	13 (4.3)	.06

Abbreviation: IQR, interquartile range.

^a^ Participant characteristics at baseline are listed for the full sample and by control group and intervention group. *P* values are for tests of difference in means between the control and intervention groups.

Gambling was common among participants, with 53.3% (160 of 300) having ever gambled and mean gambling expenditures of US $16 (IQR, US $18) in the past month (Table 1). Among all participants, 27.3% (82 of 300) reported purchasing alcohol in the past week, with slightly higher alcohol use in the control group than the intervention group (32.4% [48 of 148] vs 22.4% [34 of 152], *P* = .05). Participants spent a mean of US $2 (IQR, US $2) on alcohol in the past week. Almost half (47.3% [142 of 300]) reported exchanging money or other goods or services for sex in the past 6 months. The mean expenditures on transactional sex in the past month were US $8 (IQR, US $8) among all participants and US $20 (IQR, US $20) among those who reported any spending on transactional sex in the past month. On a weekly basis, participants’ expenditures on alcohol, gambling, and transactional sex totaled a mean of US $6, or 20% of weekly earnings. Forty-one percent (122 of 300) of participants reported at least 2 sexual partners in the past month, and 50.3% (151 of 300) perceived themselves to be at moderate or high risk of acquiring HIV in the next year.

**Figure 2.**
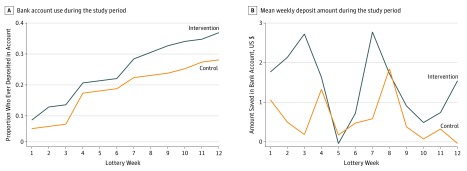
Bank Account Use by Study Group Results are shown for the mean (SD) of 9 (2) weeks that participants’ bank account balances were monitored.

During the mean 9 weeks that participants’ bank account balances were monitored, 37.3% (50 of 134) of participants in the intervention group saved any money compared with 27.2% (37 of 136) in the control group, although the difference was not statistically significant (odds ratio [OR], 1.62; 95% CI, 0.96-2.74) (Table 2). This nonsignificant difference persisted in adjusted regression models and was present throughout the study period (Figure 2). Overall, the intervention group saved approximately US $5 more than the control group during the intervention period, but the difference was not statistically significant (US $10.26; 95% CI, US $5.00-US $58.20 vs US $4.87; 95% CI, US $0.67-US $9.00; β = 5.39, SE = 5.46) (Table 2). Conditional on saving any money in the bank account, the intervention group had higher savings than the control group (US $30.40; 95% CI, US $2.90-US $58.80 vs US $21.32; 95% CI, US $5.20-US $37.40; β = 7.95, SE = 13.82).

**Table 2.** Bank Account Savings Outcomes^a^

Variable	Control Group		Intervention Group		Unadjusted Model, Odds Ratio (95% CI) or Linear Estimate β (SE)^b^	Adjusted Model, Odds Ratio (95% CI) or Linear Estimate β (SE)^b^	Adjusted and Transformed Model, Linear Estimate β (SE)^c^
	No./Total No. (%)	Mean (95% CI)	No./Total No. (%)	Mean (95% CI)			
**Binary Outcome: Ever Saved**							
Full sample (n = 270)	37/136 (27.2)	NA	50/134 (37.3)	NA	1.62 (0.96 to 2.74)	1.61 (0.93 to 2.80)	NA
High risk at baseline (n = 130)^d^	15/63 (23.8)	NA	19/67 (28.4)	NA	1.27 (0.58 to 2.79)	1.51 (0.66 to 3.46)	NA
No bank savings at baseline ^e^	20/94 (21.3)	NA	28/84 (33.3)	NA	1.85 (0.94 to 3.62)	1.85 (0.92 to 3.71)	NA
Occupation: fishing (n = 135)	18/72 (25.0)	NA	29/63 (46.0)	NA	2.56 (1.23 to 5.31)^f^	2.50 (1.18 to 5.32)^f^	NA
Occupation: transportation (n = 135)	16/64 (25.0)	NA	18/71 (25.4)	NA	1.01 (0.47 to 2.23)	0.99 (0.44 to 2.23)	NA
**Continuous Outcome: Total Amount Saved, US $**							
Full sample (n = 270)	NA	4.87 (0.67 to 9.00)	NA	10.26 (5.00 to 58.20)	5.39 (5.46)	5.68 (5.84)	0.39 (0.21)
High risk at baseline (n = 130)^d^	NA	3.85 (0.40 to 7.30)	NA	3.06 (−0.40 to 6.50)	−0.80 (2.44)	−0.74 (2.78)	0.11 (0.31)
No bank savings at baseline (n = 178)^e^	NA	2.07 (0.10 to 4.00)	NA	12.73 (−3.00 to 28.40)	10.66 (7.96)	10.78 (8.12)	0.57 (0.23)^f^
Occupation: fishing (n = 135)	NA	7.05 (−0.70 to 14.80)	NA	16.28 (−4.80 to 37.30)	9.22 (11.21)	8.89 (10.97)	0.65 (0.31)^f^
Occupation: transportation (n = 135)	NA	2.44 (0.07 to 4.80)	NA	4.93 (1.50 to 8.30)	2.50 (2.07)	2.46 (2.20)	0.18 (0.27)
**Continuous Outcome: Total Amount Saved If Savings >US $0**							
Full sample (n = 81)	NA	21.32 (5.20 to 37.40)	NA	30.40 (2.90 to 58.80)	9.53 (16.00)	7.95 (13.82)	0.26 (0.33)
High risk at baseline (n = 34)^d^	NA	18.70 (6.30 to 31.10)	NA	14.70 (5.70 to 23.70)	−3.98 (7.19)	−6.62 (9.21)	−0.66 (0.55)
No bank savings at baseline (n = 48)^e^	NA	11.90 (3.90 to 19.90)	NA	38.40 (−9.20 to 86.00)	26.47 (23.60)	23.74 (21.75)	0.46 (0.43)
Occupation: fishing (n = 47)	NA	29.60 (−0.90 to 60.20)	NA	37.30 (−8.50 to 83.10)	7.67 (26.66)	6.85 (25.12)	0.15 (0.47)
Occupation: transportation (n = 34)	NA	12.00 (3.90 to 20.00)	NA	20.50 (9.40 to 31.50)	8.49 (6.46)	10.32 (9.11)	0.46 (0.47)

Abbreviation: NA, not applicable.

^a^ Savings outcomes are from administrative bank data showing account balances at the end of each week. Analyses include all participants who registered for a bank account. Adjusted differences are coefficients from a linear regression of the outcome on treatment status, adjusting for baseline characteristics (age, marital status, less than complete primary education, occupation [fishing or transportation], weekly earnings, and had bank account at baseline).

^b^ Odds ratios (95% CIs) are given for the binary outcome and linear estimate β (SEs) for the continuous outcomes.

^c^ Data were transformed to reduce the spread in the right tail for the continuous outcome (total saved) using the inverse hyperbolic sine transformation.

^d^ High-risk men are those who reported any transactional sex in the past 6 months at baseline.

^e^ This includes those with no bank account at baseline.

^f^ *P* < .05.

Among those reporting transactional sex at baseline, there were no statistically significant differences in the proportion who saved or the total amount saved in bank accounts. Among those with no bank savings at baseline, the intervention group was more likely to save in bank accounts than the control group in the adjusted regression model, but the difference was not statistically significant (OR, 1.85; 95% CI, 0.92-3.71) (Table 2). The intervention was also more effective among fishermen, for whom the intervention group had 2.50 (95% CI, 1.18-5.32) times the odds of saving relative to the control group, whereas the intervention had no effect among participants in the transportation sector (OR, 0.99; 95% CI, 0.44-2.23).

Using survey data, we assessed outcomes of self-reported total savings (eTable 2 in Supplement 2), as well as alcohol use, gambling, and transactional sex (Table 3). Participants in the intervention group reported higher, but not significantly different, total savings from all sources at follow-up relative to control (US $201; 95% CI, US $133-US $269 vs US $145; 95% CI, US $88-US $202). At follow-up, expenditures on transactional sex in the past month were higher in the intervention group than the control group (US $16; 95% CI, US $6-US $26 vs US $9; 95% CI, US $6-US $12); these differences were not statistically significant in adjusted or difference-in-differences regression models. Spending on alcohol and gambling was similar across control and intervention groups when comparing these outcomes at follow-up and in difference-in-differences regression models. Among high-risk participants, the proportion purchasing any alcohol in the past week was 17 percentage points higher (β = 0.17, SE = 0.10) in the intervention group than the control group in the difference-in-differences model (Table 3). Among participants with no bank savings at baseline, total amounts saved were not significantly different between control and intervention groups. However, in difference-in-differences regression results, the intervention led to a US $38 (SE, US $22) increase in savings. Food and nonfood expenditures (eTable 1 in Supplement 2) were also similar between control and intervention groups.

**Table 3.** Survey Risk Behavior Outcomes^a^

Variable	Control Group		Intervention Group		Unadjusted Model, Odds Ratio (95% CI) or Linear Estimate β (SE)^b^	Difference-in-Differences Model, Linear Estimate β (SE)
	No./Total No. (%)	Mean (95% CI)	No./Total No. (%)	Mean (95% CI)		
**Binary Outcomes**						
Full sample (n = 292)						
Any alcohol, past wk	39/142 (27.5)	NA	36/150 (24.0)	NA	0.83 (0.49 to 1.41)	0.06 (0.05)
Any gambling, past mo	72/142 (50.7)	NA	83/150 (55.3)	NA	1.20 (0.76 to 1.91)	0.01 (0.05)
Any transactional sex, past mo	56/142 (39.4)	NA	56/150 (37.3)	NA	0.91 (0.57 to 1.47)	−0.00 (0.07)
High risk at baseline (n = 136)^c^						
Any alcohol, past wk	20/65 (30.8)	NA	20/71 (28.2)	NA	0.88 (0.42 to 1.85)	0.17 (0.10)
Any gambling, past mo	41/65 (63.1)	NA	48/71 (67.6)	NA	1.22 (0.60 to 2.49)	−0.02 (0.07)
Any transactional sex, past mo	36/65 (55.4)	NA	37/71 (52.1)	NA	0.88 (0.45 to 1.73)	0.04 (0.11)
No bank savings at baseline (n = 191)^d^						
Any alcohol, past wk	27/96 (28.1)	NA	27/95 (28.4)	NA	1.01 (0.54 to 1.91)	0.07 (0.07)
Any gambling, past mo	49/96 (51.0)	NA	49/95 (51.6)	NA	1.02 (0.58 to 1.80)	0.01 (0.07)
Any transactional sex, past mo	41/96 (42.7)	NA	8/95 (8.4)	NA	0.89 (0.50 to 1.59)	−0.03 (0.09)
**Continuous Outcomes**						
Full sample (n = 292)						
Alcohol expenditure in past wk, US $	NA	2.1 (0.9 to 3.3)	NA	1.9 (1.0 to 2.9)	−0.12 (0.77)	0.15 (0.73)
Gambling expenditure in past mo, US $	NA	6.4 (4.1 to 8.7)	NA	7.9 (5.4 to 10.5)	1.55 (1.74)	−1.20 (1.71)
Transactional sex expenditure in past mo, US $	NA	8.9 (6.0 to 11.8)	NA	16.0 (6.3 to 25.7)	7.06 (5.13)	7.07 (5.40)
High risk at baseline (n = 136)^c^						
Alcohol expenditure in past wk, US $	NA	2.1 (0.7 to 3.5)	NA	2.6 (0.9 to 4.3)	0.49 (1.10)	1.31 (1.09)
Gambling expenditure in past mo, US $	NA	8.2 (4.6 to 11.8)	NA	11.2 (6.8 to 15.7)	3.00 (2.88)	−2.60 (2.81)
Transactional sex expenditure in past mo, US $	NA	14.4 (9.0 to 19.8)	NA	12.0 (6.7 to 17.4)	−2.33 (3.80)	−1.74 (4.93)
No bank savings at baseline (n = 191)^d^						
Alcohol expenditure in past wk, US $	NA	1.4 (0.5 to 2.3)	NA	2.1 (0.9 to 3.2)	0.66 (0.74)	0.97 (0.73)
Gambling expenditure in past mo, US $	NA	5.1 (3.0 to 7.3)	NA	5.5 (3.2 to 7.7)	0.31 (1.59)	−0.37 (1.75)
Transactional sex expenditure in past mo, US $	NA	8.7 (5.4 to 11.9)	NA	15.4 (3.4 to 27.4)	6.71 (6.25)	6.72 (6.62)

Abbreviation: NA, not applicable.

^a^ Difference in differences are changes in the outcome from baseline. Analyses include all participants who completed the follow-up survey. Transactional sex expenditure includes money and the value of goods and services exchanged for sex in the past month.

^b^ Odds ratios (95% CIs) are given for the binary outcome and linear estimate β (SEs) for the continuous outcomes.

^c^ High-risk men are those who reported any transactional sex in the past 6 months at baseline.

^d^ This includes those with no bank account at baseline.

## Discussion

In this randomized clinical trial conducted among men in high HIV prevalence communities in Kenya, we found that a prize-linked savings intervention increased use of savings accounts but did not significantly increase the amount of money saved over a 9-week period compared with a savings account with standard interest. The intervention increased savings among the almost 70% of participants who had no bank savings at baseline, suggesting that prize-linked savings accounts may be particularly useful for motivating savings in this important population. As for health behaviors of men, we did not observe significant reductions in expenditures on alcohol, gambling, and transactional sex due to the intervention. The modest gains in savings due to the intervention suggest that more powerful savings incentives are needed to promote savings among men, and larger increases in savings may have greater potential for shifting spending away from risky behaviors.

There are several plausible explanations for the small effects of the intervention on savings behavior, which stand in contrast to prior studies^12,14^ of prize-linked savings. One possibility is that participants received insufficient information on how to deposit money into their new bank accounts. At the end of the study, many participants indicated they needed more training in making deposits through the bank’s mobile banking platform. Unlike the dominant mobile money platform in Kenya (MPesa) that 97.7% (293 of 300) of study participants reported having used, low familiarity with the banking partner’s mobile platform and its integration with MPesa may have impeded saving. Another possibility is that participants in the intervention group did not comprehend or trust the prize-linked rewards scheme, which differed from traditional savings products that offered a guaranteed interest rate. Finally, the follow-up duration of 9 weeks may have been too short for participants to learn about the rewards system and change their savings behavior. Future attempts to offer prize-linked savings accounts may require greater education about the process of saving money and earning lottery-based rewards.

Our findings are consistent with prior evidence that prize-linked savings accounts have the strongest influence among those who do not already have bank accounts.^12^ This outcome is promising considering that approximately two-thirds (197 of 300) of participants herein did not have formal bank accounts and that governments and donor organizations are expanding their efforts to increase access to financial services to currently unbanked individuals.

There remains a need to further test the potential of prize-linked savings accounts and other savings-led interventions to displace spending on high-risk behaviors in favor of savings. Study participants reported spending approximately US $6 every week on alcohol, gambling, and transactional sex, an amount equivalent to 20% of their weekly earnings. Given this high spending, the small effect of the intervention on savings may ultimately explain why the intervention failed to generate changes in men’s risk behaviors. Interventions that generate larger increases in savings would have greater potential to displace men’s spending on risky behaviors. With the growing popularity of sports betting in many parts of SSA, including Kenya, further efforts to develop and test more powerful, well-framed prize-linked savings interventions may identify new ways to reduce spending on sports betting and other forms of gambling.^12,16^ Because men who engage in risky behaviors like alcohol use, gambling, and transactional sex may have inherent preferences for risk, it is plausible that prize-linked savings products that more prominently frame saving as a way to win large monetary prizes will be particularly appealing to such men.

This is the first study to date to apply a savings intervention to target upstream HIV risk behaviors among men in SSA. There is a large literature history demonstrating that financial interventions can successfully target some HIV-related behaviors, including HIV testing^22^ and uptake of voluntary medical male circumcision.^23^ Relatedly, a recent trial in Uganda demonstrates the potential for savings interventions to improve adherence and viral suppression among HIV-positive adolescents receiving treatment.^24^ However, these studies largely focus on how economic interventions may affect use of HIV prevention or treatment services. In contrast, prize-linked savings interventions aim to affect upstream, HIV prevention–related behaviors by indirectly targeting men with preferences for risk, and they have the advantage (if successful) of averting new HIV infections by reaching men who are resistant to traditional public health messaging around primary HIV prevention.

### Strengths and Limitations

Strengths of this study include the rapid recruitment of high-risk men and the high uptake of savings accounts among participants. The study also obtained information from men on sensitive issues, such as participation in transactional sex, which has been understudied, allowing us to disaggregate the results by baseline risk behaviors of participants. The study also used a randomized clinical trial design to test the prize-linked savings intervention and relied on objective, administrative bank data to measure the primary savings outcome.

Key limitations of the study were primarily related to the small sample size and the first known attempt to implement a novel intervention in the study population. As a first study to explore the effects of a prize-linked savings intervention, the small sample size limited our ability to detect small effects. Furthermore, the bank account registration process involved many administrative steps, including registration with the Kenya Revenue Authority before the bank’s internal process for opening accounts. This delayed bank account activation for participants and shortened the overall duration of the intervention. There were additional logistical barriers to using the bank accounts, including a change in the pricing for mobile transactions that occurred early on in the study. While we obtained administrative data from the bank, other outcome measures were self-reported, putting them at risk of underreporting due to social desirability bias or recall bias. Another limitation was that lotteries were carried out weekly, and prizes were either 20% or 100% of a participant’s savings in that week. Given that most participants saved small amounts each week, the total prize amounts may not have seemed large enough to compete with gambling or other uses for participants’ money. More powerful incentives, including lotteries with larger payouts, could lead to higher rates of savings and subsequent behavior change.

## Conclusions

This randomized clinical trial provides suggestive evidence that prize-linked savings accounts can increase use of savings accounts among men at high risk for HIV in Kenya, although this intervention did not significantly differ from standard-interest savings accounts. Better-designed, more powerful incentives for savings, as well as improved intervention implementation, are necessary to assess the full potential of prize-linked savings interventions to promote future-oriented behaviors among men to increase saving and reduce spending on alcohol, gambling, and transactional sex.

## Notes

Supplement 1. **Trial Protocol**

Supplement 2. **eTable 1.** Intent-to-Treat Analysis of Main Bank Savings Outcomes **eTable 2.** Survey (Self-reported) Total Savings Outcomes

Supplement 3. **Data Sharing Statement**
