# Supplementary material for: Effect of a Prize-Linked Savings Intervention on Savings and Healthy Behaviors Among Men in Kenya: A Randomized Clinical Trial
Source: JAMA Netw Open. 2019 Sep 13;2(9):e1911162. doi: 10.1001/jamanetworkopen.2019.11162 (PMC6745050; doi:10.1001/jamanetworkopen.2019.11162)
Supplement: Supplement 1. — Trial Protocol [file jamanetwopen-2-e1911162-s001.pdf]

Prize-linked savings initiatives for promoting better health and economic outcomes in Kenya (the Akiba Study)

A study of the:

University of Pennsylvania – Impact Research and Development Organization Collaboration

Sponsored by: University of Pennsylvania

Protocol version: 2.0

Version Date: 12 July 2018

INVESTIGATORS AND INSTITUTIONAL AFFILIATIONS

Principal Investigators:	Harsha Thirumurthy, PhD<br>Associate Professor<br>Department of Medical Ethics and Health Policy<br>University of Pennsylvania<br>Associate Director, Center for Health Incentives and Behavioral Economics (CHIBE)<br>423 Guardian Drive, Blockley Hall<br>Philadelphia, Pennsylvania<br><br>Kawango Agot, PhD, MPH<br>Director<br>Impact Research and Development Organization<br>Kisumu, Kenya
Co-Investigators:	Ellen Moscoe, ScD<br>Post-doctoral researcher<br>Department of Medical Ethics and Health Policy<br>University of Pennsylvania<br><br>Dean Yang, PhD<br>Professor<br>Department of Economics, Ford School of Public Policy<br>University of Michigan<br><br>Allison Groves, PhD<br>Assistant Professor<br>Community Health and Prevention<br>Dornsife School of Public Health<br>Drexel University
Other Study Personnel:	Henry Ogola, PhD<br>Research Manager<br>Impact Research and Development Organization, Kisumu, Kenya

ABSTRACT

Despite a large decline in new adult HIV infections in eastern and southern Africa from 2005-2015, progress has slowed in recent years and multiple disparities have persisted within countries and between men and women. In particular, HIV risk among adolescent girls and young women in the region remains extremely high. Transactional sex, or the exchange of material support or goods in non-commercial sexual relationships that are often age-disparate, is widely believed to be among the main driving factors for the HIV risk in this population. Although various structural and biomedical HIV prevention interventions are have targeted adolescent girls and young women, there is a large gap when it comes to interventions targeting men who engage in transactional sex. The proposed pilot project seeks to fill this important gap by using behavioral economics principles to promote savings and shift money from high-risk activities toward saving for the future.

The project will assess the acceptability, feasibility, and potential impact of a novel prize-linked savings intervention designed to shift men's income away from alcohol and transactional sex and towards saving for the future. Prize-linked savings accounts have been in existence for many years in some countries and they offer savers a random, lottery-like payout proportional to the amount participants save, instead of traditional interest income. A number of banks, employers, and policymakers have recently promoted this low-cost, scalable approach to increasing savings among low-income individuals. However, there have been no assessments of whether prize-linked savings interventions can induce changes in not only economic behaviors like savings but also key health-related behaviors. We will conduct a pilot randomized trial among men in Kenya to assess whether offering prize-linked savings opportunities leads to reduced spending on alcohol and transactional sex. In addition, we will explore whether the intervention leads to reduced financial stress and builds future-orientation among men – all of which could lead to reduced risk-taking behavior. The project will enroll men who in communities with high HIV risk, randomize them to the savings intervention, and assess changes in key economic and self-reported health outcomes over a 3-6 month period with baseline and follow-up surveys. Findings from the pilot trial may reveal new ways to influence the HIV epidemic in priority regions where it continues unabated and will directly inform the design of a larger-scale study that focused on ways to achieve changes in health and economic behaviors.

LIST OF ACRONYMS

HIV: Human immunodeficiency virus
AGYW: Adolescent girls and young women
AIDS: Acquired immune deficiency syndrome
ATM: Automatic teller machine
PEPFAR: President's emergency program for AIDS relief
SMS: Short message service
IPA: Innovations for Poverty Action
PI: Principal investigator
IRB: Institutional review board
NIH: National Institutes of Health
USD: U.S. dollar

1.0 BACKGROUND

Transactional sex is among the main factors behind high HIV incidence in parts of eastern and southern Africa and among adolescent girls and young women (AGYW) in particular.[1-3] The intensity of the HIV epidemic in Kenya's Nyanza region – particularly in communities along Lake Victoria shorelines and islands – warrants a combination of biomedical and behavioral interventions. From the beginning of the epidemic in Africa, HIV/AIDS has been concentrated in Lake Victoria fishing communities in Kenya, Tanzania and Uganda. HIV incidence rates remain persistently high in these communities despite progress against the spread of HIV in the region overall.[3-5] Transactional sex, venue-based commercial sex work, and alcohol consumption, which occur at elevated rates in the region[4-6] are important contributors to the continued spread of HIV. The risk of HIV acquisition by AGYW is further enhanced by the age of their sexual partners being substantially higher. *This study is significant because it will explore an approach whose goal is to achieve sexual behavior change among* ***men*** *in communities with continued high rates of HIV.*

Policies to address HIV risk among AGYW have largely focused on achieving behavior change among them or providing essential services to them, but have neglected to address the role played by men's demand for transactional sex with AGYW. When men are targeted with HIV prevention services, the focus has been on increasing their uptake of voluntary medical male circumcision and HIV testing, and linking those who are HIV-positive to treatment, rather than focusing on what motivates their sexual behavior. *This study will address a missing element of prevention efforts in regions with a high prevalence of transactional sex by deploying a behavioral economics intervention to shift spending away from high-risk activities like transactional sex among men.*

The rapid expansion of mobile phone ownership in Africa has also led to increased access to financial services among the poor. In the past 10 years, mobile phone companies began to offer a "mobile money" service in which people who own a cell phone number could deposit, withdraw and transfer money simply by visiting a local "cash point" (in rural areas, this is typically a kiosk in villages). Essentially, this made it easier for individuals to save money and send money to each other, with kiosks where people typically buy vouchers to top up their phone credit serving as bank branches. Use of mobile money services is now ubiquitous in Kenya due to the early introduction of the *M-Pesa* service by Safaricom, a leading mobile phone company.[7, 8] The services were used by 96% of Kenyan households and there were 110,000 "cash points" in the country (as compared to only 2,700 bank ATMs). Mobile money is now widely used in nearly all developing countries.[9] A recent study in *Science* showed that expansion of mobile money in Kenya contributed significantly to poverty alleviation, and that it led to increased financial resilience and occupational choice.[7] This expansion of mobile phone ownership and mobile money has induced banks to provide a wider array of savings products targeted at previously unbanked individuals and communities. Many banks now offer savings accounts in which deposits and withdrawals can be made by using mobile money, rather than visiting physical branch offices of the banks.

With the widespread availability of savings accounts in Kenya and other countries, there is an opportunity to deploy novel interventions that promote public health objectives. To our knowledge, no studies have sought to use savings accounts to influence men's spending on risk factors for HIV such as alcohol and transactional sex. *We propose to use prize-linked savings accounts as a vehicle for a novel intervention that will encourage men to alter their expenditure patterns and save more money.* Our theoretical rationale is that when offered incentives to save money, men will save more of their income and spend less on alcohol and transactional sex. There are other ways in which incentivizing saving may reduce sexual risk

behavior. Lower spending on alcohol itself can reduce sexual risk-taking since alcohol is a known risk factor. Men may also increase their consideration of future health and well-being when they start to save money. Our idea builds on prior research showing that poverty alone does not explain why the poor save very little of their income. Instead, behavioral factors including a lack of incentives for saving have been noted as a key reason.[10, 11]

Behavioral economics provide a strong rationale for using lottery incentives to promote various health and economic behaviors. Insights from psychology and economics point to various factors and decision-making biases that make lottery incentives a valuable strategy for motivating individuals to adopt certain behaviors.[12] As seminal behavioral economic research of Kahneman and Tversky has shown, people tend to overestimate small probabilities,[13] implying that lotteries with low probabilities of winning large prizes are very effective in motivating individuals. Lotteries used as incentives have been successful at altering various health behaviors and they remain popular among many individuals in the U.S. and abroad. *Given the proven ability of lottery incentives and rewards to motivate individuals generally, it is likely that lottery incentives linked to savings amounts will encourage men to save a greater portion of their income on a regular basis.*

"Prize-linked savings" accounts exist in several countries and have generally succeeded in boosting individuals' savings. Prize-linked savings accounts typically offer savers a random, lottery-like payout in the form of cash or goods instead of traditional interest income.[14, 15] The probability of winning prizes is usually determined by the amount of new deposits made each month, meaning that individuals' chances of winning a prize go up as they save more each month. A recent study in South Africa showed that prize-linked savings accounts were attractive to a broad group of individuals, but *especially* to those who did not previously save money and to those with a greater tendency to take risks.[16] In South Africa, the prize-linked savings account added a fun, "gaming" element to saving money and increased total amounts saved by 38%. Based on the popularity of such accounts globally,[17] there is reason to believe that Kenyan men will have considerable interest in saving more of their income if offered a lottery-like incentives to do so. *Thus, targeting income-earning men with incentive to save money (and accordingly, disincentives to spend money on transactional sex) has as-yet untested potential to serve as an effective risk-reducing intervention among men.* This study will focus on men engaged in fishing and transport industries in communities with low savings rates. These individuals may be responsive to a savings intervention because they earn money on a daily basis and thus have cash available, a pre-requisite to saving. The structure of the prize-linked savings intervention may be especially relevant to these men, who are generally younger and whose occupations involve risk-taking, and who thus may be interested in the risky, lottery-style prizes.

2.0 JUSTIFICATION and AIMS

The proposed pilot project seeks to fill a large gap in the development and evaluation of interventions targeting the men who engage in transactional sex with AGYW and other high-risk HIV behaviors. Applying concepts from behavioral economics we will explore the acceptability and feasibility of a prize-linked savings intervention designed to encourage men to reduce spending on alcohol and transactional sex and increase savings for the future. The intervention is intended to offer men an incentive to shift their spending toward saving for the future, and therefore away from certain behaviors (like alcohol use and transactional sex) which put them at high risk for HIV acquisition and transmission. Given the promise of prize-linked savings intervention as well as the paucity of interventions that target men and seek to reduce their demand for transactional sex, the proposed pilot project has the potential to represent an

exploratory step in a new and important direction. This pilot project will generate preliminary data necessary for designing a well-powered randomized controlled trial of a savings intervention designed to reduce men's engagement in transactional sex and lower their HIV risk and the associated risk to AGYW.

The primary aim of the project is to conduct a pilot randomized trial of a prize-linked savings intervention to promote savings behavior among up to 300 high-risk men in Siaya, Kenya. We will assess changes in financial savings and expenditures on key items of interest from the perspective of HIV risk (alcohol use, gifts and transfers to sexual partners, and sexual behavior) in order to evaluate the likely effectiveness of the intervention. This data will provide preliminary information on feasibility and acceptability of the intervention in order to develop a larger trial of the prize-linked savings intervention. The project may also include a qualitative component to gain further insights on acceptability and impact of the intervention.

3.0 STUDY DESIGN

3.1 Overview of study design

We will develop a researcher-implemented prize-linked savings intervention that is linked to participants' savings account balances. We will develop a system of lottery-based rewards that participants can win depending on how much their account balance goes up (e.g. for every 100 Ksh by which savings increases, participants get an entry into a lottery in which there is a chance of winning monetary rewards). We may also consider other ways to structure rewards for saving and explore the potential for interventions to build future-orientation among men. Participants in the study will be randomized to a control group or an intervention group that receives the prize-linked savings intervention.

3.2 Study sites

The study will be conducted in communities in Siaya County, Kenya, where HIV prevalence was estimated to be 24.6% in 2014[18].A recent study[19] from Siaya County reported that adult HIV prevalence in the Gem subarea of Siaya County was 15%. This prevalence remains well above the national HIV prevalence, however, making it a priority area for this study. Siaya County is part of the populous Nyanza region, which has been the focus of Kenya's HIV prevention efforts and those of donors like PEPFAR. The high HIV incidence – particularly among AGYW – and the contribution of transactional sex to the epidemic in this region has been widely documented.[20-22]

3.3 Study population

The target population will be men who work in fishing and other informal sector jobs such as public transport and *jua-kali* (artisans).

4.0 RANDOMIZED TRIAL PROCEDURES

4.1 Recruitment

Working in ~2-5 shoreline communities in Siaya County, trained research assistants will identify potential participants and will conduct a short set of screening questions to determine eligibility prior to obtaining informed consent. These questions will obtain information on men's occupation since existing research indicates that those who earn income on a regular basis (fishermen, transportation worker, and other laborers) are more likely to have multiple sexual partnerships, including some that have a transactional element. Eligible individuals will be recruited immediately following the screening questions. We will seek to enroll about 300 men who meet study eligibility criteria.

4.2 Eligibility criteria
This study will focus on an intervention aimed at changing savings and expenditure patterns, and risk behaviors, among men in communities with high prevalence of transactional sex. Thus we will exclusively recruit men in the pilot phase.

Inclusion criteria:

1. Resident in selected shoreline communities in Siaya County, Kenya
2. Male
3. Age 21 years and above
4. Primary or secondary occupation is fishing or transportation (i.e. motorbike taxi driver)
5. Owns mobile phone
6. Is willing to open a savings account with a local bank

Exclusion criteria:

1. Planning to relocate from study communities in the next 6 months
2. Does not express willingness to open a savings account

4.3 Consent and enrollment processes
Participants eligible for enrollment in the study will be approached by the study team at the time of the baseline survey and will complete an informed consent procedure immediately prior to the baseline survey. The informed consent form will be distributed to potential participants and will be read aloud (in English, Swahili or Dholuo depending on participant's preference). Survey staff will respond to questions and offer further explanations where necessary, and will ask participants to sign the consent form (or to make a mark, in the case of respondents who cannot write.) For those desiring more time to make a decision, survey staff will leave the consent document with the potential respondent and will follow up on a subsequent day.

Following enrollment in the study, a baseline questionnaire will be administered to all participants (described below). All participants will then be asked to open a savings account with a local banking partner, a process that the study will facilitate. There will be no out-of-pocket cost for participants to open an account and there will be no requirement to deposit their own money into the account.

4.4 Randomization procedures
Participants will be randomly assigned to either an intervention or control group using simple random assignment. Individuals meeting eligibility criteria and providing consent will be randomized in equal proportions to one of two groups, a control group or an intervention group that receives the prize-linked savings intervention.

4.5 Intervention group
Research assistant will explain the structure of the lottery prizes to participants randomized to the lottery incentive group. Participants will be told their chance of winning monetary rewards will be based on the amount by which their account balance goes up (e.g. for evert 100 Ksh by which savings increases, participants get an entry into a lottery for monetary rewards.) This type of prize-linked savings intervention has been shown to promote savings in other settings. During the intervention period, participants' savings account activity will be checked at regular intervals and lottery prizes will be determined at these intervals. Participants will learn about the lottery outcome via SMS and prizes will be deposited directly into their savings accounts.

Other intervention components may include education materials to explain how the prize-linked savings incentives work and that emphasize the potential benefits of saving money (having greater ability to cope with setbacks, build or upgrade their homes, pay school fees, buy farm

inputs, buy more motorbikes for use in business, or start a different business; etc.). Participants in the intervention group will be encouraged to have more consideration for their future health and economic status, as this may motivate them to save more money. They will also be encouraged to consider the opportunity and health cost of their expenditures on alcohol and transactional sex and not miss the opportunity to win prizes by saving money.

4.6 Comparison group

During the study period, participants' savings account activity will also be tracked at regular intervals, but they will not be eligible to receive lottery-style prizes. They may instead receive interest on the amount they have saved at a standard rate that is determined by the banking partner.

4.7 Data collection activities

Structured questionnaires will be administered to all participants at the time of enrollment (baseline) and at study completion. Data will be collected by a team from Innovations for Poverty Action (IPA), which has led a number of household surveys in the Nyanza region. Trained research assistants from IPA will administer these questionnaires using tablet computers. Participants will receive a small amount of compensation for the time required to complete each survey.

Baseline data collection: A baseline questionnaire will collect information on demographic characteristics, employment and income, assets and savings, consumption and expenditures, self-reported sexual behavior, alcohol and substance use, HIV risk perceptions, and questions about financial stress, future orientation, and expectations. Sexual behavior questions will inquire about past engagement in transactional sex (defined broadly to include financial and non-financial transfers from the partner to the woman, approximate age of sexual partners).

Interim data collection: Additional contact from the survey team may occur at regular intervals during the intervention period. These intermittent contacts may occur via SMS or in person and are expected to take only a few minutes.

Endline data collection: An endline survey with similar content as the baseline will be administered about 3-4 months after study enrollment in order to measure economic and health outcomes that have the potential to be affected by the intervention..

The questionnaire modules will be based on questionnaires that have been used in other studies in the Nyanza region and on surveys such as the Demographic and Health Survey and the World Bank's Living Standards Measurement Surveys.

Qualitative interviews: In addition to the baseline and endline surveys, we may conduct in-depth interviews with up to 20 men to explore perceptions of the prize-linked savings intervention. Beyond exploring the extent to which the intervention motivated men to save money and change their health and economic behavior, the interviews will also assess other factors that influence men's decisions – including barriers to saving such as beliefs about mortality risks in the future.

4.8 Outcomes

We will be interested in assessing four primary outcomes:

1. Increase in savings balance: This is a binary variable equal to 1 if the respondent's savings balance increased over the study period, and zero otherwise.
2. Change in savings balance over full study period: Similar to outcome 1, but a continuous measure of the net increase or decrease in savings balance over the study period.
3. Expenditures on alcohol

4. Participation in transactional sex
5. Expenditures on transactional sex

We will additionally assess the following secondary outcomes:

1. Savings: Net increase in both the savings balance and in self-reported total savings from all sources, in each lottery period and over the entire study period.
2. Expenditures on food items
3. Expenditures on non-food items

4.9a Data analysis
We will use intent-to-treat analyses to compare outcomes between the intervention and control groups at endline. Our analyses will include subgroup analyses by baseline risk behavior level. We will perform linear or logistic regressions to estimate the effect of the intervention on each outcome of interest, and will include control variables for selected baseline characteristics and for all variables used in randomization.

4.9b Sample size and power calculations
Because this is a pilot study, we may not have sufficient power to detect statistically significant effects on all of the primary outcomes. We aim to recruit a sample size sufficient to detect a 15 percentage point increase in probability of increasing the savings balance (outcome 1), with 80% power and alpha=0.05. The necessary sample size will be 200 participants if we assume that 10% of participants in the control groups will have an increase in savings between baseline and endline. With a sample size of 300 participants and 50% of participants in the control group having an increase in savings, we will by 80% power to detect a difference of ≥16 percentage points in the probability that savings increases due to the intervention.This pilot project is intended to assess the feasibility and acceptability the prize-linked savings intervention and will also generate vital preliminary data on likely effect sizes on important economic and health outcomes. It will also aid in the development of recruitment procedures and data collection tools that will be used for a larger scale-up of the intervention if the pilot results are promising. As such, we will balance the need for a large enough sample to detect statistically significant changes in our primary outcome with the need to keep the pilot study at a small scale.

5.0 ETHICAL CONSIDERATIONS

5.1 Potential risks to human subjects
The risks to study participants are minimal as the project primarily involves baseline and follow-up survey questions and an intervention to promote financial savings. For some participants there is a possibility of experiencing distress from being asked to answer personal survey questions about sexual behavior.

5.2 Adequacy of protection against risks

5.2.1 Informed consent
Informed consent will be obtained from all participants by trained research assistants. Because some participants may not be able to read at a level enabling them to understand the consent form, the survey team will offer to read the letter in its entirety and will discuss the consent process in detail verbally. The research assistants will find a private place; carefully explain the nature and purpose of the study to each potential participant, potential risks and benefits, compensation for participation, and will describe the two study groups to which participants will be randomized. The consent process will be offered in English, Kiswahili, or Dholuo (as per the

participant's preferences). The banking partner will also obtain consent from respondents to share their savings data with study staff. Our study team has prior experience in obtaining informed consent for research and clinical trials within the cultural context of in Kenya. For those who cannot sign the informed consent form, they will be asked to make a mark with the pen or to use an inkpad and make a thumbprint indicating their choice to participate, and a witness will also sign indicating that free and informed consent was given. The informed consent procedure has been designed to maximize understanding of potential risks. Participants will be told that they may decline to participate at any point. The consent procedure will be carried out at baseline, prior to administering the baseline survey. For those who consent, the survey will take place immediately after the consent procedure. For those who are unsure, the survey team will attempt to answer their questions and will work with the potential respondent to find another time on a subsequent day for a follow-up visit.

In order to protect against risks associated with answering sensitive questions, all participants will be reminded that they are not obligated to answer any survey questions that they do not feel comfortable answering.

5.2.2 Maintaining privacy and avoiding stigmatization

Participants will be interviewed during the day, primarily near the locations where they work. Interviewers will make every effort to find a secluded location where others cannot overhear the interview. Prior to any questionnaire modules that ask about sexual behavior or other potentially sensitive information, participants will be reminded that they may skip certain questions if they do not wish to answer.

5.2.3 Data security

We will develop standard operating procedures for data security and confidentiality procedures at collection, transfer, entry and storage levels, and make these readily accessible to all staff members who have access to confidential study data.

Individual study identifiers will be assigned to participants at enrollment. Data will not be able to be used to identify individuals directly. To protect personal information (name, phone number), data collected in surveys will be encrypted and de-identified.

All study tablets used for data collection will be encrypted and password protected. They will be autonomously monitored so that the tablet can be wiped clean in the case of a tablet going missing. All electronic study data will be stored on a secure server with access limited only to authorized staff. All computers and servers will be encrypted and password-protected with limited access.

Physical data collection forms will be stored at IPA offices and will be kept under lock and key by the Study Coordinator. They will be accessible only to the PIs and Coordinator. A key linking identification numbers of respondents to the respondent identity will be kept in file separate from the main dataset, will only be accessed by key study personnel, and will be encrypted. This key will be destroyed once data collection is complete. At the end of the study, any data containing identifying information will be kept for up to 5 years for electronic version and up to 2 years for paper forms, including consent forms.

5.2.4 Institutional Review Board approval

Approval from the University of Pennsylvania institutional review board and the Maseno University institutional review boards (IRBs) will be obtained prior to initiation of any study activities. All study staff are required to undergo training in human subjects research, and good clinical practice.

5.3 Potential benefits of the proposed research to the participants and others

Participants may directly benefit from the study: a small number of participants will have the opportunity to win prizes if they choose to save money over the course of the study. Prizes will be allocated at random among the set of participants who increase their savings during the study. Participants may also experience benefits from increased savings even if they do not win prizes.

Participants will contribute to a body of knowledge about effective interventions to increase savings and to reduce HIV risk-related behaviors among men. This can indirectly benefits those who may be targeted by future interventions based on this body of evidence, including adolescent girls and young women who face high HIV risk stemming from age-disparate relationships and transactional sex.

5.4 Importance of the knowledge to be gained

Findings from the pilot trial may reveal new ways to influence the HIV epidemic in priority regions where it continues unabated and will directly inform the design of a larger-scale study that focused on ways to achieve changes in health and economic behaviors. Several aspects of this study would fall directly within the HIV prevention priorities laid out by NIH and countries in eastern and southern Africa. The project has potential to identify sustainable and scalable ways to promote better economic and health outcomes.

6.0 LIMITATIONS

We recognize potential critiques of the proposed project and will attempt to address these to the best of our ability. A primary limitation in our study is that it is a small pilot project, therefore we may not be able to answer all questions with a high level of statistical significance and we may not be able to perform many sub-group analyses due to sample size limitations. This will be resolved if we proceed to a future scaled-up study. In addition, some of our outcomes (such as sexual behavior) will be self-reported. This is not a major problem, however, because we will obtain these measures in both study groups so we can compare changes among and between the groups.

7.0 DISSEMINATION OF RESEARCH FINDINGS

The results will be shared with the study site staff and other stakeholders in relevant forums. The results will also be presented at local and international meetings and conferences where other researchers and policymakers are present, particularly those working on HIV prevention and treatment. In addition, we will prepare manuscripts and submit them to peer-reviewed journals for publication and wider dissemination. No individual identities will be used in any reports or publications resulting from the study.

8.0 STUDY TIMELINE

	May	June	July	Aug	Sept	Oct	Nov	Dec	Jan	Feb
Protocol prep., IRB submission	x									
Meetings with local partners in Kenya		x	x							
Hire and train research assistants				x						
Study community selection & mobilization				x						
Recruit & enroll study participants				x	x					
Baseline data collection				x	x					
Intervention implementation				x	x	x	x			
3-mth follow-up survey data collection							x	x		
Analysis and dissemination									x	x

9.0 BUDGET

**Category**	**Amount (USD)**
Project personnel	14621.13
Travel	5809.47
Census and Baseline Survey	4593.22
Endline Survey	4283.97
Supplies	1500.45
Intervention costs	404.39
Other direct costs	2720.23
Subtotal	33932.86
Overhead (15%)	5089.92
**Total**	**39022.78**

10.0 ROLES AND RESPONSIBILITIES

The study is being conducted by Drs. Harsha Thirumurthy and Kawango Agot.

Title	Role	Responsibilities
Co-PI<br><br>Co-PI	Dr. Harsha Thirumurthy<br><br>Dr. Kawango Agot	Dr. Thirumurthy will lead study design, execution, analysis, and presentation of study data<br><br>Dr. Agot will co-lead study design, execution, and oversee study implementation. She will also contribute to interpretation of results and dissemination.
Co-Investigators	Dr. Ellen Moscoe<br><br>Dr. Dean Yang<br><br>Dr. Allison Groves	Dr. Moscoe will contribute to study and intervention design, and will take a leading role in analysis and manuscript writing.<br><br>Dr. Yang will contribute to measurement of outcomes and assist in results interpretation and dissemination.<br><br>Dr. Groves will contribute to measurement of transactional sex and other sensitive questions in the surveys.
Other Study Personnel	Dr. Henry Ogola	Dr. Ogola will provide local support for the study including periodic site visits and participation in conference calls.

11.0 References

1. U.N.A.I.D.S, *HIV prevention among adolescent girls and young women*. 2016, New York: UNAIDS.
2. Global, U.N.A.I.D.S., *AIDS Update*. 2016, New York: UNAIDS.
3. Kiwanuka, N., et al., *High Incidence of HIV-1 Infection in a General Population of Fishing Communities around Lake Victoria, Uganda.* PLoS One, 2014. **9**(5): p. 94932.
4. Seeley, J., J. Nakiyingi-Miiro, and A. Kamali, *High HIV incidence and socio-behavioral risk patterns in fishing communities on the shores of Lake Victoria, Uganda.* Sex Transm Dis. **2012**(39): p. 433-9.
5. Seeley, J.A. and E.H. Allison, *HIV/AIDS in fishing communities: Challenges to delivering antiretroviral therapy to vulnerable groups.* AIDS Care, 2005. **17**(6): p. 688-97.
6. Camlin, C.S., Z.A. Kwena, and S.L. Dworkin, *Jaboya vs. jakambi: Status, negotiation, and HIV risks among female migrants in the "sex for fish" economy in Nyanza Province.* AIDS Educ Prev, 2013. **25**(3): p. 216-31.
7. Suri, T. and W. Jack, *The long-run poverty and gender impacts of mobile money.* Science, 2016. **354**(6317): p. 1288-92.
8. Suri, T., W. Jack, and T.M. Stoker, *Documenting the birth of a financial economy.* Proc Natl Acad Sci, 2012. **109**(26): p. 10257-62.
9. G.S.M.A, *State of the Industry Report: Mobile Money*. 2015.
10. Dupas, P. and J. Robinson, *Why Don't the Poor Save More? Evidence from Health Savings Experiments.* American Economic Review, 2013. **103**(4): p. 1138-71.
11. Banerjee, A.V. and E. Duflo, *The Economic Lives of the Poor.* Journal of Economic Perspectives, 2007. **21**(1): p. 141-68.
12. Volpp, K.G., et al., *P4P4P: an agenda for research on pay-for-performance for patients.* Health Aff (Millwood), 2009. **28**(1): p. 206-14.
13. Kahneman, D. and A. Tversky, *Prospect theory: an analysis of decision under risk.* Econometrica, 1979. **47**(2): p. 263-91.
14. Kearney, M., et al., *Making Savers Winners: An Overview of Prize-Linked Savings Products.* NBER Working Paper No 1643, 2010.
15. Filiz-Ozbay, E., et al., *Do lottery payments induce savings behavior? Evidence from the lab.* Journal of Public Economics, 2015. **126**: p. 1-24.
16. Cole, S., B. Iverson, and T.P. Can, *Can Gambling Increase Savings? Empirical Evidence on Prize-Linked Savings Accounts.* Saïd Business School WP 2014-10, 2017.
17. Tufano, P., J.E. De Neve, and M.N. U.S, *Consumer demand for prize-linked savings: New evidence on a new product.* Economics Letters, 2011: p. 111-2.
18. Council, K.N.A.C., *Kenya AIDS Response Progress Report*. 2014, Kenya.
19. Borgdorff, M.W., et al., *HIV incidence in western Kenya during scale-up of antiretroviral therapy and voluntary medical male circumcision: a population-based cohort analysis.* The Lancet HIV, 2018. **5**(5): p. e241-e249.
20. Camlin, C.S., et al., *“She mixes her business”: HIV transmission and acquisition risks among female migrants in western Kenya.* Social Science & Medicine, 2014: p. 102-0.
21. Cassels, S. and C.S. Camlin, *Geographical mobility and heterogeneity of the HIV epidemic.* The Lancet HIV, 2016. **3**(8): p. e339-41.
22. Kwena, Z., I. Mwanzo, and C. Shisanya, *Predictors of Extra-Marital Partnerships among Women Married to Fishermen along Lake Victoria in Kisumu County, Kenya.* PLoS One, 2014. **9**(4): p. e95298.
