# Supplementary material for: Effect of a Prize-Linked Savings Intervention on Savings and Healthy Behaviors Among Men in Kenya: A Randomized Clinical Trial
Source: JAMA Netw Open. 2019 Sep 13;2(9):e1911162. doi: 10.1001/jamanetworkopen.2019.11162 (PMC6745050; doi:10.1001/jamanetworkopen.2019.11162)
Supplement: Supplement 2. — eTable 1. Intent-to-Treat Analysis of Main Bank Savings Outcomes eTable 2. Survey (Self-reported) Total Savings Outcomes [file jamanetwopen-2-e1911162-s002.pdf]

Supplementary Online Content

Moscoe E, Kawango A, Thirumurthy H. Effect of a prize-linked savings intervention on savings and healthy behaviors among men in Kenya: a randomized clinical trial. *JAMA Netw Open*. 2019;2(9):e1911162. doi:10.1001/jamanetworkopen.2019.11162

eTable 1. Intent-to-Treat Analysis of Main Bank Savings Outcomes

eTable 2. Survey (Self-reported) Total Savings Outcomes

This supplementary material has been provided by the authors to give readers additional information about their work.

© 2019 Moscoe E et al. *JAMA Network Open*.

eTable 1. Intent-to-Treat Analysis of Main Bank Savings Outcomes

	Control (N=148)	Intervention (N=152)	Unadjusted model	Adjusted model	Adjusted and Transformed
Ever saved, N (%) and OR (95% CI)	34 (23%)	47 (31%)	1.50 (.90-2.51)	1.46 (.86-2.48)	-
Total saved, mean [95% CI] and B (SE)	4.48 [0.62-8.35]	9.05 [0.27-17.83]	4.57 (0.35)	4.61 (0.36)	0.32 (.19)*

Analyses include full sample. Outcomes for 30 participants who did not register for bank accounts are set to zero for intention-to-treat analysis. Adjusted models show are coefficients from a linear or logistics regression of the outcome on treatment status, adjusting for baseline characteristics (marital status, less than primary education, occupation (fishing or motorbike taxi), had bank account at baseline, weekly earnings, and age. Transforming outcomes uses the inverse hyperbolic sine transformation to reduce spread in the right tail of the distribution of outcomes. Savings outcomes are from administrative bank data showing account balances at the end of each week.
***$p<0.01$, **$p<0.05$, *$p<0.1$.

© 2019 Moscoe E et al. *JAMA Network Open*.

eTable 2. Survey (Self-reported) Total Savings Outcomes

	(1)	(2)	(3)	(4)	(5)
Outcome: Total savings (US$)	**Control Mean [95% CI]**	**Intervention Mean [85% CI]**	**Unadjusted model β (SE)**	**Difference-in-differences, β (SE)**	**Difference-in-differences and transformed#, β (SE)**
Full sample (N=292, 142 control, 150 intervention)	145 [88-202]	201 [133-269]	56 (44)	57 (36)	0.30 (0.18)
High-risk at baseline[1] (N=136, 65 control, 71 intervention)	186 [75-299]	195 [98-293]	9 (74)	15 (51)	0.46 (0.25)*
No bank savings at baseline[2] (N=191, 96 control, 95 intervention)	106 [44-169]	114 [73-156]	8 (38)	38 (22)*	0.35 (0.23)
Occupation: Fishing[3] (N=148, 74 control, 74 intervention)	177 [69-286]	270 [144-396]	92 (83)	90 (66)	0.17 (0.28)
Occupation: Transportation[4] (N=144, 68 control, 76 intervention)	110 [86-133]	135 [81-188]	25 (29)	29 (30)	0.42 (0.23)*

Savings outcomes are from the baseline and endline surveys and include all forms of savings, both formal and informal. Cols 1-2 show mean [95% CI], cols 3-5 show coefficient with standard error. Difference-in-differences estimates are changes in the outcome from baseline relative to changes in the control group. Analyses include all participants who completed the follow-up survey. #Data transformed to reduce the spread in the right tail for the continuous outcome (total saved), using the inverse hyperbolic sine transformation. *p<0.1, **p<0.05, ***p<0.01

[1]High-risk men are those who reported any transactional sex in the past 6 months at baseline.

[2]No bank account at baseline includes those who reported that they had no money saved in a bank account at baseline.

[3]Participants whose main occupation is in the fishing industry

[4]Participants whose main occupation is in the transportation industry

© 2019 Moscoe E et al. *JAMA Network Open*.
