# Supplementary material for: Effect of a Prize-Linked Savings Intervention on Savings and Healthy Behaviors Among Men in Kenya: A Randomized Clinical Trial
Source: JAMA Netw Open. 2019 Sep 13;2(9):e1911162. doi: 10.1001/jamanetworkopen.2019.11162 (PMC6745050; doi:10.1001/jamanetworkopen.2019.11162)
Supplement: Supplement 3. — Data Sharing Statement [file jamanetwopen-2-e1911162-s003.pdf]

Data Sharing Statement

Moscoe. Effect of a Prize-Linked Savings Intervention on Savings and Healthy Behaviors Among Men in Kenya. *JAMA Netw Open*. Published September 13, 2019. 10.1001/jamanetworkopen.2019.11162

Data
Data available: Yes
Data types: Deidentified participant data
How to access data: Data set will be uploaded to https://dataverse.harvard.edu/
When available: With publication

Supporting Documents
Document types: None

Additional Information
Who can access the data: Anyone requesting the data
Types of analyses: For any purpose
Mechanisms of data availability: Data will be available to download without restriction
